# Cadmium, Lead, Copper and Zinc in Breast Milk in Poland

**DOI:** 10.1007/s12011-013-9870-x

**Published:** 2013-12-12

**Authors:** Anna Winiarska-Mieczan

**Affiliations:** Department of Bromatology and Food Physiology, University of Life Sciences in Lublin, Akademicka 13, 20-950 Lublin, Poland

**Keywords:** Breast milk, Infant, Cadmium, Lead, Zinc, Copper, Intake

## Abstract

Mother's milk is the fundamental food for infants. It contains proteins, fat, carbohydrates and essential metals which are necessary to ensure correct functioning of the organism. Unfortunately, breast milk is a potential source of toxic metals, which are dangerous for a baby. In Poland, previous research concerning the content of metals in breast milk was very scarce or its results were unavailable. The present study aimed at assessing the content of Cd, Pb, Cu and Zn in human breast milk, as well as estimating the mean weekly intake of these metals by breast-fed infants from Poland. The average concentrations of Cd, Pb, Cu and Zn were 2.114 μg/l, 6.331 μg/l, 0.137 mg/l and 1.623 mg/l, respectively. The admissible levels of supply of these toxic metals has not been exceeded, but their contents were high, particularly in 6-month-old infants (nearly 85 % TWI for Cd and nearly 70 % BMDL_01_ for Pb). The daily intake of Cu and Zn did not fully satisfy the infant's requirements determined by Polish standards and WHO recommendations. Since the lifestyle of lactating women has a direct influence on the content of these elements in breast milk, women should be educated in this respect with particular focus on eliminating tobacco smoking, both by breastfeeding mothers and by their direct environment.

## Introduction

Breastfeeding is the best method of nutrition for infants. The World Health Organisation (WHO) recommends breastfeeding as the exclusive method of nutrition for newborns and infants at least up to 6 months of age [1]. Previous research by this author [2] carried out in the region of Lublin (eastern Poland) demonstrated that as many as 87 % of women were breastfeeding for at least 6 months. The components of mother's milk, such as lactoferrin, lysozyme and α-lactalbumin, create a barrier protecting the baby against harmful environmental factors, enhancing the body's defence mechanisms and stimulating the development of the immune system [3]. Breast milk also has an influence on the development of intestinal microflora [3] and structural and functional maturity of mucous membranes and reduces the risk of allergies and autoimmune diseases [4]. However, mother's milk can also be a source of cadmium (Cd) and lead (Pb) for a baby. The content of these elements in breast milk reflects the level of environmental pollution and the mother's diet [5–8]. The presence of these metals in food products has become a global problem [5, 9, 10]. In Poland, the highest content of Cd and Pb is found in food derived from plants including but not limited to vegetables, in particular, potatoes, and grains and cereals [11]. Mothers who smoked tobacco create an additional source of toxic trace elements in the body, thus increasing the content of Cd and Pb in the breast milk [12, 13]. Research by Öhrvik et al. [14] revealed that Cd leads to a dysfunction of the mammary glands in mice, which is the reason for developmental disorders among sucklings. Since ‘heavy metals’ is a poor scientific term [15], in the present study, the term ‘toxic metals’ is used with reference to Cd and Pb, and ‘essential metals’ with reference to copper (Cu) and zinc (Zn).

The cycle of metals in the environment is linked with the food chain: soil–plant–animal–man. The transfer of toxic metals to the higher link results in a cumulative increase in their content [16]. Cd is primarily deposited in the liver and kidneys, but in infants, it poses the greatest hazard to their dynamically developing nervous system, also affecting bone-formation processes, and is a significant carcinogen [17–20]. Tests on rats demonstrate that Cd supplied with milk affects the serotonin level in the brain of a growing animal [21]. The highest Cd accumulation levels are recorded during the first 3 years of human life [22]. In children, it can inhibit intellectual development and causes anaemia and rickets [23]. Significantly, a positive correlation between the concentration of Cd and Pb in children and the occurrence of autism has been observed [16]. Neurological effects of Pb observed in children include motor skills disorder and behavioural problems [24]. Exposure to this metal for several years after birth is particularly detrimental to the future intellectual potential of children [2, 25]. Children are exposed to accumulation of Pb due to a slower excretion process and lower body weight and also because of reduced immunity [26].

Essential metals, such as Cu and Zn, are necessary to ensure the correct functioning of the body but, in excessive amounts, have a harmful effect [27]. Cu is an element necessary for synthesising haemoglobin, forming myelin sheaths in the nervous system, and for bone tissue formation processes [27, 28]. Zn is an important element at the foetal stage [27]. A deficit of this element can lead to defects in the nervous system and cause retardation of the foetus' growth and development [29].

In Poland, previous research concerning the content of Pb, Cd, Cu and Zn in breast milk was very scarce or its results were unavailable. No detailed evaluations of the effect of age and lifestyle of Polish women on the content of metallic elements in breast milk are available. The study is aimed to evaluate the content of Cd, Pb, Cu and Zn in their breast milk and to estimate the intake of these metals by breastfed infants.

## Materials and Methods

### Materials

Breast milk samples (~25 ml) were collected by manual expression from 320 healthy lactating women from the Lublin region (eastern Poland) during 7 days of sampling. All women were informed about the purpose of the study and voluntarily consented to provide breast milk samples for analysis. Contact with the subjects was established during the medical check-up of their children in paediatric clinics.

The samples were collected in the period from August to December 2010. Each sample was sealed in a polyethylene bag and frozen at -20 °C for further analysis.

### Methods

The breast milk samples were shaken manually before the analysis. Also, three samples were taken (2 ml) from each of the bags. Samples were dried at 65°C for 12 h, next at 105°C for 24 h, and mineralised in a muffle furnace. The samples were treated by dry mineralisation at a temperature of 450°C for 12 h, and the ash obtained was suffused with 2 ml of hydrogen peroxide, evaporated until dry and once more burnt at a temperature of 450°C for 12 h. Ashed samples were diluted in spectrally pure 1 M HNO_3_. The content of Cd and Pb was determined by GF AAS technique in a Varian Spectr AA 880 apparatus, including atomisation in a graphite furnace and using the Zeeman background correction. Argon was used as the pure gas. The content of Cd was determined at λ = 228.8 nm, with 4 mA and 0.5 nm spectral bandpass (LOD 0.01 mg/kg, LOQ 0.02 mg/kg). The deviation of duplicate measurement was below 5.3 %. The content of Pb was determined at wavelength λ = 217.0 nm, with 10 mA and 1 nm spectral bandpass (LOD 0.209 mg/kg, LOQ 0.419 mg/kg). The content of Zn and Cu was determined using the FAAS flame technique in a Varian SpectrAA 280 FS apparatus (with SPS3 autosampler). Zn was detected at λ = 213.9 nm, with 5 mA and 1 nm spectral bandpass (LOD 0.01 mg/kg, LOQ 0.02 mg/kg), Cu at λ = 324.8 nm, with 4 mA and 0.5 nm spectral bandpass (LOD 0.013 mg/kg, LOQ 0.016 mg/kg). In order to make the calibration line, standard solutions of Cd, Pb, Zn and Cu were procured from Merck (Germany). A series of solutions of varying concentrations were prepared for all the examined ions by diluting the standards: 0.00, 0.10, 0.20, 0.40, 1.00 and 2.00 ng/ml. Quality control of analytical measurements was performed using blank samples and certified reference materials: IRMM-804 Rice Flour (Cd) and BCR-063R Skimmed Milk Powder (Pb, Zn and Cu). The certified reference materials contained Cd 1.16 mg/g, Pb 0.815 mg/g, Zn 49.0 μg/g and Cu 0.602 μg/g. The mean recovery rate of Cd from the reference materials was 96 %, of Pb 95 %, of Zn 117 % and of Cu 121 %.

### Calculation of Metals Intake

The mean intake of Cd, Pb, Cu and Zn in breast milk was calculated for infants aged 1, 3, 6 and 12 months. Since it is difficult to calculate the daily intake of milk by a breast-fed infant, the supply of powdered milk, as recommended in Poland, was used as a reference value [30]. The calculation of the tolerable intake of toxic metals was based on mean infant body weight (Table 1). The tolerable weekly intake (TWI) for Cd was determined at 2.5 μg/kg of body weight/week [31]. The benchmark dose lower confidence limit (BMDL_01_) for Pb was determined at 0.5 μg/kg of body weight/day (3.5 μg/kg of body weight/week) [32].

**Table 1:** Average body weight of infants from Poland and daily milk consumption

	Age of children			
	1 month	3 months	6 months	12 months
Average body weight^a^ relevant for Poland, g	4,500	6,250	7,750	10,500
Daily consumption of milk^b^, ml	700	780	900	660

^a^The average body weight relevant for Poland was estimated according to a centile chart for infants. The values for boys and girls were averaged

^b^The supply of milk formulas, as recommended in Poland, was used as a reference value, adopter from Ref. [30]

### Statistical Analysis

The results were analysed using statistical methods. Arithmetic mean values, standard deviation (SD), median, maximum and minimum values and percentile values (25 and 75) were calculated using STATISTICA 6.0 software. The *p* values < 0.05 were considered significant. Full factorial analysis of variance (ANOVA) was used to test for significant effects of the women's age, stage of lactation and tobacco smoking on Cd, Pb, Cu and Zn levels in breast milk. The significance of differences between mean values was estimated using Tukey's test.

### Profile of the Analysed Population of Women

During the first meeting the women were interviewed to determine the profile of the studied population (age, number of lactations, period of current lactation). The questions also concerned exposure of the women to factors likely to affect the content of toxic metals in milk (occupational exposure, smoking). The data was presented in Table 2.

**Table 2:** The profile of the analysed population of women

	Age of mothers, years				Total
	20–25	26–30	31–35	36–40	
Number of women	35	158	70	60	323
Place of residence					
Village	25	59	30	9	123
City < 25,000 habitants	10	28	22	0	60
City > 25,000 habitants	0	71	18	51	140
Number of lactation					
I	35	143	25	10	213
II	0	15	45	0	60
III	0	0	0	40	40
IV	0	0	0	10	10
Lactation stage					
1 month	0	40	10	9	59
2–3 months	11	52	9	36	108
4–6 months	14	36	36	0	86
7–12 months	10	30	15	15	70
Smoking habit					
Current smoker	0	20	20	0	40
Past smoker	0	23	0	3	26
Never	35	115	50	57	257
Passive smoking					
Yes	12	34	8	7	61
No	23	124	62	53	262
Occupational exposure to Cd or Pb	0	0	0	0	0

## Results

### Content of Cd and Pb in the Breast Milk

Table 3 presents the content of Cd, Pb, Cu and Zn covered by the research in the breast milk and effects of women's age. The average content of Cd in the milk slightly exceeded 2 μg/l (0.215–7.355), while that of Pb amounted to 6.3 μg/l (0.486–12.01). The highest content of Cd was found in the breast milk of women aged 31 to 35 (more than 3 μg/l), while most Pb was discovered in the breast milk of women aged 36 to 40 and 26 to 30 (approx. 7–7.4 μg/l). The lowest content of both Cd and Pb was characteristic of the milk of women aged 20 to 25.

**Table 3:** Content of Cd, Pb, Cu, and Zn in the breast milk and effect of the mother's age, μg/l

Age of mothers, years	*n*	Cd	Pb	Cu	Zn
20–25	35	0.723^a^	4.762^a^	0.088^a^	0.701^a^
26–30	158	2.151^b^	6.932^c^	0.154^c^	2.504^c^
31–35	70	3.031^d^	6.221^b^	0.137^b^	1.674^b^
36–40	60	2.552^c^	7.411^d^	0.169^d^	1.612^b^
Mean		2.114	6.331	0.137	1.623
Standard deviation		2.112	4.614	0.092	1.763
Minimum value		0.215	0.486	0.025	0.043
Maximum value		7.355	12.01	0.455	8.160
Median		1.260	1.951	0.106	1.240
25th percentile		0.557	1.602	0.069	0.448
75th percentile		3.017	9.188	0.158	1.941
Effects of women's age		*p* < 0.05	*p* < 0.05	*p* < 0.05	*p* < 0.05

^a,b,c,d^In the same column, values with different superscripts differ significantly (*p* < 0.05)

**Fig. 1:**
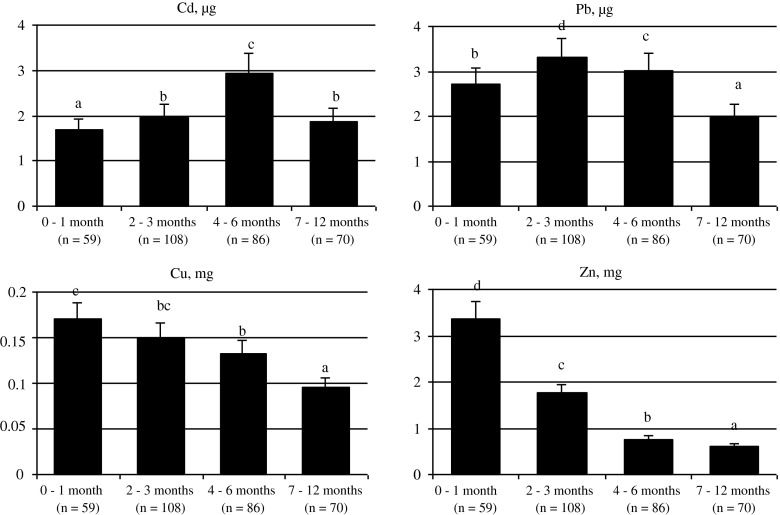
Effects of the lactation stage (months) on Cd, Pb, Cu and Zn levels in breast milk, per 1 L. ^a,b,c,d^ *p* < 0.05

The stage of lactation had a significant effect on the content of Cd (ANOVA; *F* = 9.76; *p* < 0.05) and Pb (ANOVA; *F* = 2.35; *p* < 0.05) in the breast milk (Fig. 1). The highest content of Cd was characteristic of breast milk between 4 and 6 months of lactation (2.94 μg/l), while the lowest value was recorded for the first month's milk, i.e. the colostrum (less than 1.7 μg/l). The highest content of Pb was found in milk between 2 and 3 months of lactation (nearly 3.3 μg/l), while it was definitely lower in milk between 7 and 12 months of lactation (less than 2 μg/l).

**Fig. 2:**
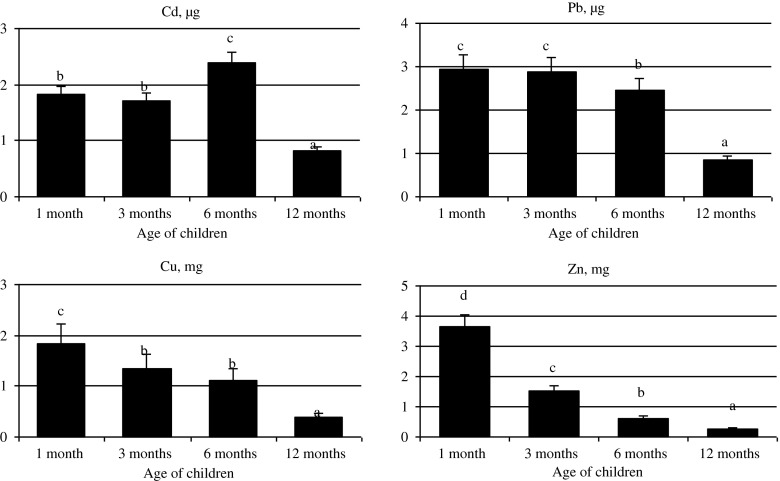
Mean weekly intake of Cd, Pb, Cu and Zn from breast milk, per 1 kg of infant's body weight. ^a,b,c,d^ *p* < 0.05

Figure 2 presents the mean weekly intake of metals by the infant in the first, third, sixth and 12th months of life per 1 kg of body weight. The results were calculated based on the mean content of the analysed components in milk, depending on the stage of lactation. The body weight of an infant and the estimated daily intake of milk is presented in Table 1. Table 4 compares the % TWI and % BMDL_01_ for infants aged 1, 3, 6 and 12 months with the average body weight recorded in Poland, assuming the value of 2.5 μg Cd [31] and 3.5 μg Pb [32] per 1 kg of body weight/week. It was determined that the admissible levels of Cd and Pb supply were not exceeded; they were elevated though. For Cd, this was from 32.8 % TWI (in 12-month-old babies) to 84.8 % TWI (in 6-month-old infants), whereas for Pb from 23.9 % BMDL_01_ (in 12-month-old babies) to 84 % BMDL_01_ (in 1-month-old infants).

**Table 4:** % TWI (for Cd) and % BMDL_01_ (for Pb) for infants aged 1, 3, 6, and 12 months with the average body weight relevant for Poland (see Table 1), μg/kg of body weight/week

	Cd	Pb
EFSA norm	2.5 μg [31]	3.5 μg [32]
Infant's age	% TWI	% BMDL_01_
1 month	72.72^b^	84.00^c^
3 months	67.70^b^	82.37^c^
6 months	84.84^c^	69.91^b^
12 months	32.84^a^	23.89^a^

^a,b,c^In the same column, values with different superscripts differ significantly, *p* < 0.05

**Fig. 3:**
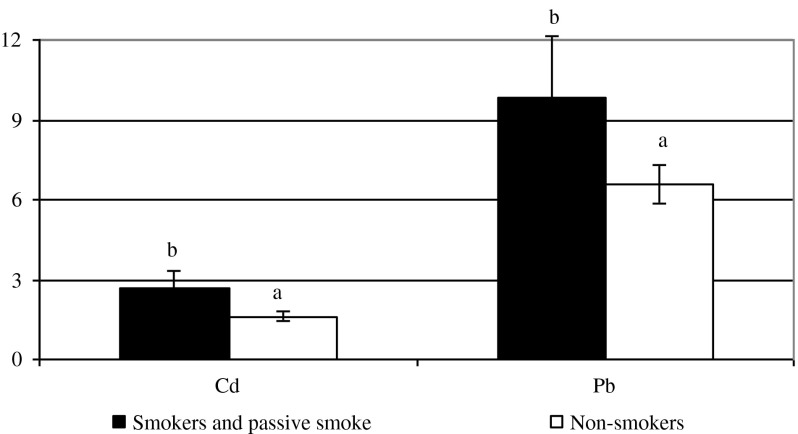
Mean levels of Cd and Pb in breast milk of smoking and non-smoking women, μg/l. ^a,b^ *p* < 0.05

Figure 3 presents the content of Cd and Pb in the breast milk of smoking women compared to non-smokers. ANOVA indicated that this factor had a significant effect on the levels of Cd (*F* = 27.08; *p* < 0.05) and Pb (*F* = 18.22; *p* < 0.05). As expected, more toxic metals were found in the milk of women who smoked tobacco.

### Content of Cu and Zn in the Breast Milk

The average content of Cu in the milk slightly exceeded 0.137 mg/l (0.025–0.455), while that of Zn amounted to 1.623 mg/l (0.043–8.160; Table 3). The highest content of Cu was recorded in the milk of women aged 26–30 and 36–40 (approx. 0.15–0.17 mg/l). The breast milk of women aged 20–25 was found to contain half as much Cu. The highest content of Zn was characteristic of the breast milk of women aged 26–30 (more than 2.5 mg/l), while the lowest value was recorded for the milk of mothers aged 20–25 aged (slightly above 0.7 mg/l).

The content of Cu and Zn in breast milk was analysed depending on the stage of lactation (Fig. 1). The highest content of Cu was recorded in the breast milk of women in the first month of lactation (0.17 mg/l), while the lowest value of this element was noted in milk between 7 and 12 months of breastfeeding (less than 0.1 mg/l). The highest content of Zn was also found in the first month's milk (more than 3.3 mg/l), while the lowest value was recorded for milk between 7 and 12 months of lactation (approx. 0.61 mg/l).

The calculated % of coverage of the infant's requirement of Cu and Zn supplied in mother's milk is presented in Table 5. It was revealed that the daily supply of Cu did not fully cover the infant's demand for this element determined in the Polish standard [33]. About 60 % of the infants' requirement was covered during the first 6 months of life compared with less than 30 % during the second 6 months. Breast milk in the first month of lactation was rich in Zn–the supply of this element exceeded the infant's requirement by approximately 17 %. Afterwards, the concentration of this element decreased and after the first 6 months of life mother's milk covered only 20 % of the Zn requirement.

**Table 5:** The calculated % of coverage of an infant's requirement of Cu and Zn from breast milk

	Cu	Zn
Polish norm, mg/day [33]	0.2–0.3	2.0
	% of Polish norm	
Infant's age		
1 month	59.39^b^	117.6^d^
3 months	60.18^b^	68.52^c^
6 months	61.69^b^	34.43^b^
12 months	29.11^a^	20.13^a^
WHO norm, mg/day [40]	0.33–0.62	5.33–5.6
	% of WHO norm	
Infant's age		
1 month	36.00^bc^	44.36^d^
3 months	36.47^c^	25.86^c^
6 months	33.35^b^	22.21^b^
12 months	9.701^a^	7.189^a^

^a,b,c,d^In the same column, values with different superscripts differ significantly, *p* < 0.05

## Discussion

### Content of Cd, Pb, Cu and Zn in the Breast Milk

The content of Cd and Pb in breast milk is a global problem. It is primarily related to the common presence of these toxic metals in the environment and thus inevitable exposure to their effect [26, 34]. The present study showed no excessive admissible levels of toxic metals supply. However, the high content of Cd and Pb in milk consumed by infants at the age of 6 months is a disturbing fact since breast milk is not the only source of these metals for them. Research revealed that toxic metals are also present in other infant food, both in Poland [35] and in other countries [36, 37]. Admittedly, the studies referred above revealed no excessive standard limits; still, there is a risk of excessive supply of Cd and Pb in infants fed with breast milk and, at the same time, given complementary foods, particularly when in 2012 the admissible intake levels of Cd and Pb were radically reduced [31, 32]. In Table 6, the levels of Cd and Pb in breast milk recorded in this study were compared with those of other selected countries. The recorded Cd milk levels were considerably lower than those reported in Spain, Brazil and Nigeria and were higher than those of women from Sweden and Italy. The highest content of Cd—within the range of 0.6–1.3 μg/l—was recorded in the milk of mothers from Spain. The content of Pb resulting from the present study was comparable to data from Austria—the results referred to the maximum levels of Pb determined for this country—and was significantly lower than in women from Spain, Iran, Saudi Arabia and China (Table 6).

**Table 6:** Cd, Pb, Cu and Zn concentrations in breast milk of women from Poland and from other countries

	Cd, μg/l	Pb, μg/l	Cu, mg/l	Zn, mg/l	References
Poland	0.21–7.35	0.49–12.0	0.03–0.46	0.04–8.16	This study
Poland	5.4				[60]
Poland			0.27–0.45	1.4–8.2	[48]
Slovakia	0.43	4.7			[61]
Sweden	0.07	0.5			[62]
Sweden	0.028–0.27	0.74–6.4	0.32–0.67	1.24–5.71	[50]
Spain	0.6–11.3	0.1–32.3			[63]
Austria		0.02–11.17			[64]
Austria	0.22–0.26				[65]
Portugal		0.07–4.03	0.33–0.97	0.39–5.09	[47]
Croatia			0.27–1.35	0.62–15.0	[38]
Greece	0.19	0.48	0.38	4.90	[46]
Brazil	1.0–8.0				[34]
Nigeria	9.7	8.7	0.83	0.7	[66]
Kuwait			0.41–0.71	1.7–3.2	[67]
Japan	0.07–1.22		0.12–0.70	2.73–11.6	[51]
Japan			0.35	1.45	[68]
Libya			0.39–0.84	4.95–16.1	[39]
Iran	0.62–6.35	3.18–24.67			[13]
Iran	0.45–5.87	3.06–19.47			[69]
Iran			0.36	2.95	[44]
Saudi Arabia	1.73	31.67	3.10		[70]
Guatemala			0.09–0.60	0.47–6.19	[71]
China	0.67	40.6			[72]
Italy	<LOQ	0.85–1.07	0.35–0.42	0.70–0.90	[73]

Mandić et al. [38] found that the breast milk of Croatian women contained nine times more Zn than the milk analysed during the present study (Table 6). The content of Zn in the analysed samples amounted to 1–4.95 mg/l. The highest content of Zn was recorded in the breast milk of women from Libya and the lowest in women from Venezuela [39]. According to Hannan et al. [39], the daily intake of Cu and Zn from the Libyan mothers' milk is higher than the recommended daily amount (RDA) value, whereas this study shows that the daily supply of Cu and Zn did not fully satisfy the infant's demand for those elements as defined by the Polish standard [33] and the WHO standard [40]. A low level of Cu causes no proven risk of nutritional deficiencies of Cu in the child during the first 6 months of lactation. Babies are born with hepatic reserves of Cu to balance low concentrations of these elements in breast milk [41]. Additionally, Zn and Cu in breast milk are characterised by high bioavailability compared with infant milk formulas [42–44], and it may be supposed that the body's resources of these trace elements are sufficient for breast-fed babies. On the other hand, a low content of Cu is a condition for bacteriostatic properties of breast milk [45]. However, it must be remembered that mother's milk is the sole source of food for an infant only during the initial 5–6 months of life, so a deficiency of Cu and Zn after the first 6 months of life should not cause alarm since the infant is also supplied with this element in other food.

### Effects of Lactation Stage

The present study findings suggest a relationship between the stage of lactation and the content of Cd and Pb in milk. Leotsinidis et al. [46] found that the colostrum of Greek women contained much more Cd and Pb (0.19 and 0.48 μ/l) than the post-colostral milk (0.14 and 0.15 μ/l). By contrast, in our study, the lowest value for Cd was recorded for the first month's milk.

The content of Zn and Cu in breast milk depends on the stage of lactation. Almeida et al. [47] recorded 500 μg (0.5 mg) of Cu and nearly 2300 μg (2.3 mg) of Zn per 1 l of breast milk on the 30th day of lactation. According to Wasowicz et al. [48], the highest content of Zn and Cu is found in colostrum. The present study also demonstrated that the milk of mothers in the first 6 months of lactation contained more Zn and Cu than in the second 6 months. A statistical analysis carried out by Rydzewska and Król [49] revealed a correlation between the concentration of Zn and the day of the postpartum period and increased content of Zn and reduced level of Cu in the colostrum. Leotsinidis et al. [46] found that the colostrum of Greek women contained much more Zn than the post-colostral milk (4.9 vs. 2.99 mg/l). However, no significant difference was found in the content of Cu in the colostrum and the post-colostral milk. A different content of essential trace elements in the colostrum and the post-colostral milk can occur due to genetic conditions of the lactation process [50].

### Effects of Women's Age

According to Rahimi et al. [13] the average concentration of Cd and Pb in women aged under 30 years was lower than in women aged over 30 years. In this study, the lowest content of both Cd and Pb was characteristic of the milk of women aged 20 to 25. The present study shows that the age of the women is also a factor affecting the concentration of essential trace elements in milk. Honda et al. [51] show that a woman's age is a factor affecting the concentration of essential trace elements in her breast milk. Picciano [52] found that the breast milk of women over 30 contains more Zn and Cu than that of women under 30. By contrast, Koyashiki et al. [34] and Khaghani et al. [44] found no statistically significant effect of the woman's age on the content of Zn and Cu.

### Effects of Lifestyle

Cd and Pb in breast milk reflected maternal exposure. Nishijo et al. [7] observed a positive correlation between maternal Cd burden and Cd concentration in breast milk. Gundacker et al. [6] found a correlation between the content of Pb in the breast milk of Austrian women and their body weight, duration of pregnancy as well as diet and lifestyle. These authors have found the highest accumulation of Pb in the milk of women weighing less than 60 kg and women who gave birth after week 37 of pregnancy. Also the content of this element was significantly higher than in the milk of women who often consumed fish and food supplements.

Smoking had a great influence on the Pb value in breast milk. The milk of women who have never smoked tobacco contained 1.57 μg of Pb, while the milk of currently smoking women–2.4 μg/l (5). Research by Kwapuliński et al. [53] revealed the highest content of Cd in the breast milk of active and passive smokers—0.27 μg/g. In the group of non-smokers, the content of Cd was lower—approximately 0.20 μg/g. The present study also showed that the content of Cd and Pb in the milk of women who smoked was significantly higher than in non-smokers. According to Rahimi et al. [13], the concentration of Cd and Pb in breast milk significantly increased in mothers who were exposed to smoking compared to non-smokers (3.39 vs. 2.12 μg/l of Cd and 12.22 vs. 9.78 μg/l of Pb). The present study showed that the content of Cd and Pb in the breast milk of women who smoked tobacco was significantly higher than in non-smoking mothers.

Many researchers found no influence of Cu and Zn in the diet on the content of Cu and Zn in breast milk [54, 55], while single researchers demonstrated that the level of Zn slightly increased after receiving Zn supplements [56]. Domellöf et al. [57] found no correlation between the content of Cu and Zn in milk and the mineral status of a woman. On the other hand, however, since breast milk of women from different regions of the world, who due to climatic, economic and cultural differences have different diet routines, contains different amounts of Zn and Cu (Table 6), the effect of the diet routine on the content of these essential metals in milk cannot be completely excluded. It is possible that a diet deficient in Zn and Cu has an influence on the low content of these elements in milk. Hambidge [29] found that Zn deficiency was a problem affecting approximately 50 % of the population. Some authors found that the diet of Polish girls and women is deficient in Zn [58]. According to Szymelfejnik et al. [59], 50 % of female students are at a high risk of Zn deficiency and approximately 90 % are likely to have Cu deficiency.

To sum up, the admissible levels of Cd and Pb supply were not exceeded but their content was high, particularly in 6-month-old infants (nearly 85 % TWI for Cd, nearly 70 % BMDL_01_ for Pb). It is significant that the analysed milk contained insufficient amounts of Zn and Cu compared with an infant's requirements of these elements. The reasons for low levels of these essential metals in breast milk must be carefully reviewed. Perhaps these could be attributed to nutritional mistakes of lactating women. The analysed milk should be regarded as safe for infants; however, as breast milk is the basic food for infants, it must be continuously monitored for the level of toxic and essential metals. Since the lifestyle of lactating women has a direct influence on the content of these elements in breast milk, women should be educated in this respect with particular focus on eliminating tobacco smoking, both by breastfeeding mothers and by their direct environment.
